# Hub Genes and Key Pathways of Intervertebral Disc Degeneration: Bioinformatics Analysis and Validation

**DOI:** 10.1155/2021/5340449

**Published:** 2021-09-10

**Authors:** Zhiwen Zhang, Qiong Wang, Yang Li, Bangzhi Li, Liming Zheng, Chengjian He

**Affiliations:** ^1^College of Acupuncture and Orthopedics, Hubei University of Chinese Medicine, Wuhan 430061, China; ^2^Hubei Provincial Hospital of Traditional Chinese Medicine, No. 4, Garden Hill, Wuchang, Wuhan, Hubei 430061, China

## Abstract

**Objective:**

To identify significant pathways and genes in intervertebral disc degeneration (IDD) based on bioinformatics analysis.

**Design:**

The GEO database was used to download the GSE124272 dataset. Differentially expressed genes (DEGs) were analyzed using Limma package in R language. Then, gene ontologies (GO), Kyoto encyclopedia of genes and genomes (KEGG), and protein-protein interaction (PPI) networks were used to further identify hub genes. The mRNA expression levels of top six hub genes were verified.

**Results:**

We found 563 DEGs, of which 214 were upregulated and 349 were downregulated. The top 5 GO terms and pathways were shown including immune response, cell cycle, and p53 pathway. Based on the PPI analysis, we verified the mRNA expression levels of 6 hub genes. The mRNA levels of CHEK1, CDCA2, SKA3, and KIF20A were upregulated in degenerative NP tissue than in healthy NP tissue. However, the mRNA level of BUB1 and SPC25 was downregulated.

**Conclusions:**

This study may provide new biomarkers for the IDD and treatments to repair IDD related to CHEK1, CDCA2, SKA3, BUB1, KIF20A, and SPC25.

## 1. Introduction

Intervertebral disc degeneration (IDD) is a significant cause of intervertebral disc degeneration diseases including low back pain, stenosis, lumbar disc herniation, and ischialgia, which can cause the worldwide economic and social burden and seriously affect quality of life [1–8]. Boden et al. found that all subjects between 60 and 80 years of age showed IDD, though 35% of subjects between 20 and 39 years of age showed at least one lumbar level of IDD [9]. There is currently no effective treatment for IDD to reverse and repair IDD [10]. In recent decades, finding effective treatments and developing appropriate treatment procedures for IDD have become the focus of research [11–17].

The anatomical structure of a complete intervertebral disc consists of the surrounding annulus fibrosus (AF), the central nucleus pulposus (NP), and the cartilage endplate (CEP) [18]. During IDD, inflammation, oxidative stress, apoptosis, senescence, and other pathological factors are involved. IDD is considered a result of multifactorial contributions including trauma, inflammation, age-related changes, and local nutritional and vascular dysfunction [19]. At present, conservation and surgical treatments are often used in the treatment of IDD, but those methods can only relieve symptoms and may recur repeatedly, limiting spinal activity [20]. One key reason for the current situation is the lack of a clear understanding of its pathophysiology and molecular mechanisms [21].

Hub genes are defined as having the highest degree of connectivity, suggesting functional importance in the diseases. Thus, investigating the hub genes and key pathways associated with IDD is necessary to clarify the pathophysiology and molecular mechanisms, which provides potential effective therapeutic strategies.

Recently, microarray technology and bioinformatics analysis have become popular methods of exploration of disease pathogenesis and identification of biomarkers for disease progression [22]. The purpose of this study is to identify potential molecular targets and signaling pathways associated with IDD based on Gene Expression Omnibus datasets. To identify potential hub genes among these DEGs, we constructed protein-protein interaction (PPI) networks. These hub genes were also validated using human nucleus pulposus (NP) samples, which reveals potential molecular mechanisms associated with IDD.

## 2. Research Materials and Methods

### 2.1. Retrieving Data

The GEO database (http://www.ncbi.nlm.nih.gov/geo) is a gene expression database created by NCBI (the National Biotechnology Information Center of the United States). This GSE124272 dataset was downloaded from the GEO database, which consisted of 16 whole blood samples from 8 patients with intervertebral disc degeneration and 8 patients with healthy discs. This dataset was published by Wang Yi et al. [23]in 2019, and the patients were genotyped using the GPL21185 Agilent-072363 SurePrint G3 Human GE v3 8x60K Microarray.

### 2.2. Data Processing and Identification of DEGs

We used affy package (https://bioconductor.org/biocLite.R) in the R software bioconductor to read the data. The robust multiarray averaging (RMA) algorithm was used to normalize the data. Finally, we used the Limma package (Limma package R 3.4.3) to identify DEGs. *P* < 0.05 and |log_2_FC | ≥1 were used to identify DEGs from these samples, and volcano plot was constructed.

### 2.3. Functional Enrichment Analysis of DEGs

The DAVID (http://david.abcc.ncifcrf.gov/) database is mainly used for functional enrichment analysis of DEGs [24]. We used the DAVID database to enrich and analyze the functions and pathways including gene ontology (GO) terms and Kyoto encyclopedia of genes and genomes (KEGG) terms with a significant threshold of *P* < 0.05. GO terms consisted of three categories: biological process (BP), cellular component (CC), and molecular functional (MF).

### 2.4. PPI Analysis

PPI networks were constructed to predict protein-protein interactions of DEGs using the STRING database (http://www.stringdb.Org). Then, these data were uploaded into Cytoscape software (https://cytoscape.org/) to visualize the networks of DEGs. Finally, we used MCODE plug-in in Cytoscape to identify the hub genes based on the previously constructed PPI networks.

### 2.5. Verification of Hub Genes

We obtained nucleus pulposus (NP) tissues from two patients with acute lumbar disc herniation or degenerative disc disease. One patient underwent posterior lumbar interbody fusion, and another underwent percutaneous endoscopic lumbar discectomy. The relatively healthy NP tissues were grades I~II, and degenerated NP tissues were grades III~V according to Pfirrmann classification score by magnetic resonance imaging. The Pfirrmann grades of these two NP tissues, respectively, are grade II and grade V. The NP tissues were harvested under sterile conditions and immediately sent to the laboratory. Written informed consent was obtained from each patient. The study was approved by the Ethics Committee of Hubei Provincial Hospital of Traditional Chinese Medicine.

### 2.6. RNA Extraction and Quantitative Real-Time- (qRT-) PCR

We used TRIzol reagent (Ambion, Foster City, CA, USA) to extract total RNA from human NP tissues according to the manufacturer's instructions. We used PrimeScript RT Master Mix (Takara Bio, Shiga, Japan) to reverse transcribe total RNA according to the manufacturer's instructions. Then, qRT-PCR was performed using the One-Step SYBR PrimeScript RT-PCR Kit (Takara Bio) to quantify the mRNA expression levels of SPC25, CHEK1, CDCA2, SKA3, BUB1, and KIF20A. Endogenous housekeeping gene GAPDH was used to normalize these mRNA expression levels. The 2^−*ΔΔ*Ct^ method was used to compute these relative expression levels.  Table 1 shows the primer sequences used for qRT-PCR.

### 2.7. Statistical Analysis

SPSS statistical software 24.0 was used for statistical analysis (SPSS, Inc., Chicago, IL, USA). Each experiment was carried out at least three times. The results are performed as the mean ± standard deviation (SD). These data were analyzed using Student's *t*-test. To evaluate the difference in gene expression level between two groups, *P* < 0.05 was considered significant.

## 3. Results

### 3.1. Identification of DEGs

**Figure 1:**
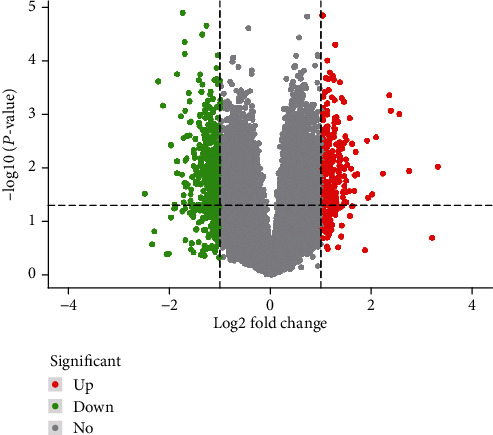
Volcano plot of DEGs. Red represents high expression, green represents low expression, and black represents no difference.

We analyzed the DEGs between two groups in the GSE124272 based on *P* < 0.05 and ∣log_2_FC | ≥1 and found 563 DEGs, of which 214 were upregulated and 349 were downregulated. Volcano plot was shown to visualize the DEGs in Figure 1.

### 3.2. GO and KEGG Functional Enrichment Analysis of DEGs

**Figure 2:**
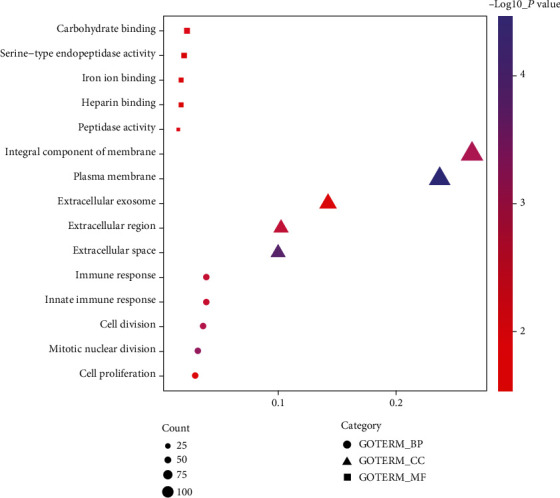
Top five enriched GO terms associated with the DEGs.

**Figure 3:**
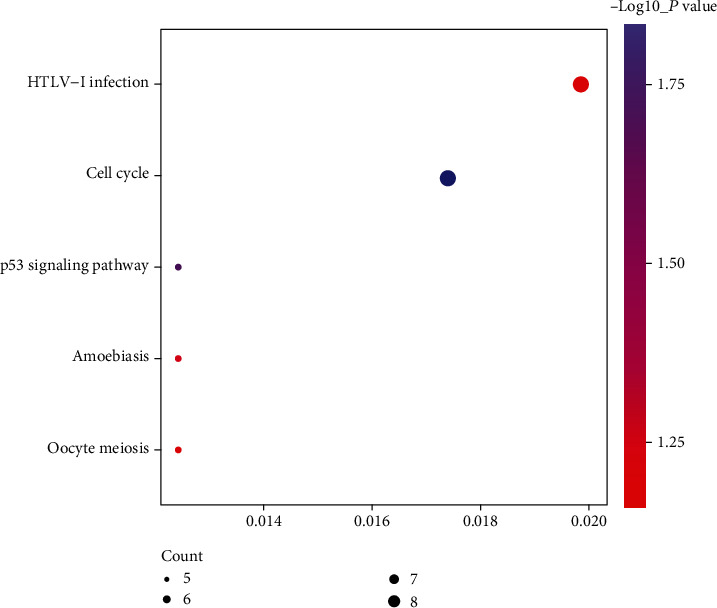
Top five enriched KEGG terms associated with the DEGs.

We analyzed the functional enrichment of DEGs to explore the potential molecular mechanism and related genes. The top five enriched GO terms and the KEGG pathway of DEGs are shown in Figures 2 and 3 and Table 2.

### 3.3. PPI Network and Hub Gene Analysis

**Figure 4:**
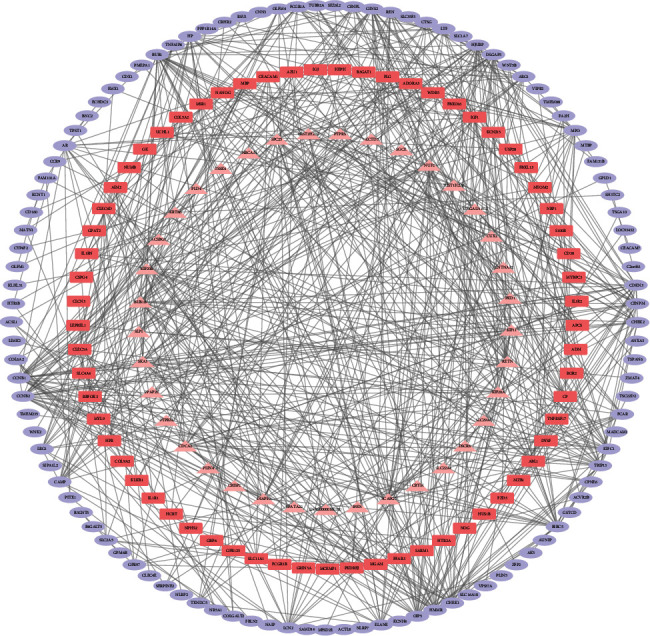
Protein-protein interaction (PPI) network of DEG.

**Figure 5:**
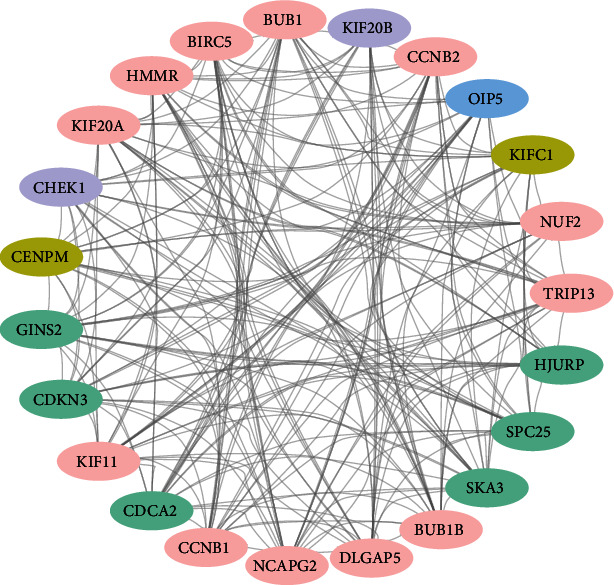
Top 20 hub genes identified by PPI.

In order to identify the potential interaction between the DEGs, we built a PPI network and used the Cytoscape software to visualize it. In the intervertebral disc degeneration group, the PPI network included 207 nodes and 668 edges (Figure 4), and the top 20 hub genes were identified by MCODE (Figure 5).

### 3.4. Validation of the Hub Genes

**Figure 6:**
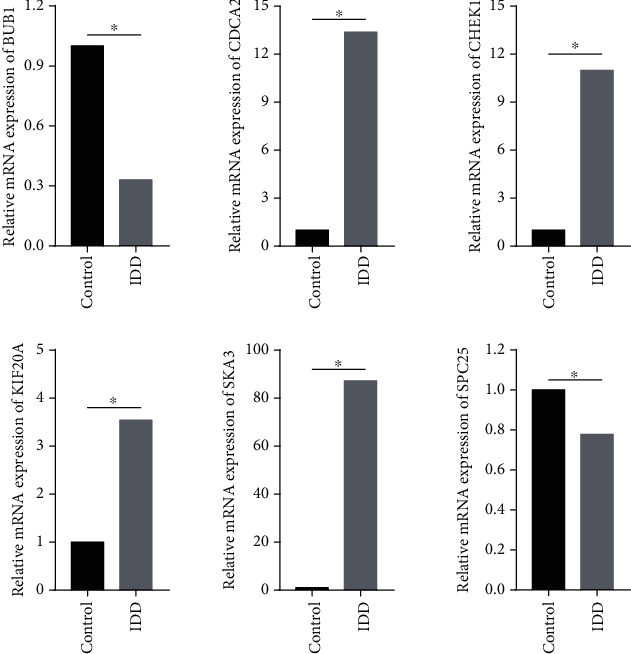
Validation of the mRNA expression levels of the top six hub genes.

To validate the identified hub genes, we used two human NP tissues including one from a patient with healthy disc and one from a patient with degenerated disc to identify the mRNA levels of the top six hub genes. The mRNA levels of CHEK1, CDCA2, SKA3, and KIF20A were upregulated in degenerative NP tissue than in healthy NP tissue. However, the mRNA level of BUB1 and SPC25 was downregulated in degenerative NP tissue. These results are shown in Figure 6.

## 4. Discussion

IDD plays an important role in spine-related diseases. It is difficult to reverse the IDD progression with current treatments. Although numerous studies have studied the mechanisms of IDD, the underlying mechanisms remain unclear. In our study, the GSE124272 was used to identify the DEGs of two groups. GO and KEGG enrichment analysis were performed to find significant biological processes and signal pathways. The important biological processes are related to immune response, innate immune response, cell division, mitotic nuclear division, and cell proliferation. It is worth mentioning that cell proliferation plays an important role in IDD. Many studies have confirmed that the proliferation level of intervertebral disc cells is diminished during IDD progression. On the contrary, the apoptosis rate of intervertebral disc cells increases relatively [25–31]. Many molecules regulate this process of IDD, including proteins, microRNAs, and long noncoding RNAs [25, 32–40]. Wang et al. [33] demonstrated that lncRNA-RMRP promoted NP cell proliferation and upregulated expression of cyclin D1 and PCNA.

KEGG pathway analysis showed several pathways associated with IDD. p53 signaling pathway have been widely studied in IDD [27, 41–44]. Many biological processes are involved in p53 pathway, including cell proliferation, apoptosis, and senescence. p53 is a transcription factor and well-known as a tumor suppressor in humans and other mammals, which participates in the regulation of biological processes. It has been reported that the p53 pathway regulates the senescence process of cartilage end plate cells and p53 phosphorylation level can been decreased through silencing of SUMO2 [27].

PPI network analysis was used to identify hub genes in IDD. We validated six hub genes including CHEK1, CDCA2, SKA3, BUB1, SPC25, and KIF20A. Cell division cycle associated 2 (CDCA2) is a cell-related protein, which is related to CDCA1, 3, and 4-8 [45]. It has been reported that upregulation of CDCA2 regulated by HIF-1*α* inhibited apoptosis and promoted proliferation in prostate cancer [46]. HIF-1*α*, a hypoxia-inducible factor, is a transcriptional factor which affects the homeostatic maintenance of NP tissue and extracellular matrix metabolism [47]. Uchida et al. [48] confirmed that silencing of CDCA2 significantly inhibited cellular proliferation and promoted apoptosis. In addition, CDCA2 performs regulation function through signal pathways including the MAPK pathway [49].

SKA3, a subunit located in the kinetochore outer layer of the SKA complex, performs biological function related to the NDC80 complex to affect proper mitotic exit during mitosis, which regulates cell proliferation and migration [50]. In CRC cells, silencing of SKA3 reduced cell growth rates and increased apoptosis, inducing G2/M arrest and decreasing migration, and anchorage-independent growth [51]. Accumulating evidence indicates that SKA3 induces the expression of matrix metalloproteinase- (MMP-) 2, MMP-7, and MMP-9 via activating the PI3K/AKT signal pathway which regulates numerous cellular functions mainly including angiogenesis, metabolism, cell growth, cell proliferation, protein synthesis, transcription activity, and cell apoptosis [52, 53].

It is found that SPC25 plays an important role in regulating cell proliferation, apoptosis, and invasion. Cui et al. demonstrated that knockdown of SPC25 suppressed cell proliferation through decreasing in the number of cells in the S phase and increasing in the number of cells in the G2/M phase [54]. Chen et al. [55] demonstrated that SPC25 was upregulated in lung cancer tissues and was involved in the regulation of tumor cell proliferation and metastasis. Additionally, SPC25 knockdown upregulated expression level of p53, indicating that the p53 signaling pathway is a potential pathway associated with SPC25 [56]. p53, a cellular stress sensor, responds to diverse stress signals including DNA damage, hypoxia by regulating cell senescence, and apoptosis in the intervertebral disc [57].

Kinesin-like family member 20A (KIF20A), a mammalian mitotic kinesin-like motor protein of the Kinesin superfamily proteins, is related to Golgi apparatus dynamics and considered a significant molecule for cell cycle regulation. It is also found that KIF20A regulates the localization of subset of central spindle components [58]. A further study indicated that KIF20A affected the process of porcine early embryo development.

Zhao et al. [59] demonstrated that KIF20A can promote tumor cell proliferation and inhibit apoptosis in vivo and in vitro. Likewise, Duan et al. [60] confirmed that cellular proliferation and invasion were promoted through upregulation of KIF20A, and cell viability and invasion capacity were inhibited via silencing of KIF20A. These findings indicate that KIF20A may be a novel target associated with NP cell degeneration.

Checkpoint kinase 1 (CHEK1), a serine/threonine-specific protein kinase, regulates the DNA damage response and cell cycle checkpoint reactions and plays a significant role in the S and G2 cell cycle checkpoints [61, 62]. In order to inhibit damaged cells from developing throughout the cell cycle, CHEK1 is activated to affect the initiation of cell cycle checkpoints, cell cycle arrest, DNA repair, and cell death, which regulates the phosphorylation level of several downstream effectors to trigger a pleiotropic cellular response [63]. Upregulation of CHEK1 is involved in various types of cancer and promotes tumor progression via affecting cell cycle and DNA damage response including breast cancer, pancreatic cancer, and oral squamous cell carcinoma [64]. It has been demonstrated that upregulation of CHEK1 can ameliorate the overall survival of non-small-cell lung cancer patients and miR-195 downregulates the expression level of CHEK1, which inhibits cell migration, growth, or invasion [65].

BUB1, a component of the spindle assembly checkpoint which is a surveillance mechanism of ensuring genome stability by delaying anaphase, is involved in cell divisions through regulating SAC function and yielding a highly aberrant mitosis. Accumulating evidence indicates that centromeres in BUB1-deficient cells separate prematurely, which reveals that BUB1 is essential for the proliferation of fibroblasts. Schliekelman et al. found that the upregulation level of BUB1 promoted tumorigenesis and had oncogenic properties [66–68]. Likewise, Zhu et al. [69] demonstrated that BUB1 overexpression promoted liver cancer cell proliferation and downexpression of BUB1 suppressed cell proliferation by activating the phosphorylation level of SMAD2. It has been reported that SMAD2 regulates NP cell proliferation and anabolic processes by activation of TGF-beta 1. Additionally, silencing of BUB1 activates a p53-dependent premature senescence response, which induces IDD through cell cycle arrest, cellular senescence, or apoptosis in the intervertebral disc [70].

In conclusion, we have used bioinformatics analysis to identify CHEK1, CDCA2, SKA3, BUB1, SPC25, and KIF20A as hub genes related to IDD, which provided a new insight into IDD pathogenesis and treatment. Further studies should be performed to verify these results.

## Figures and Tables

**Table 1:** Primer sequences.

Gene	Sequence	Size
CDCA2	Forward: 5′-TGTGGGCAGCTCTGTAGAAA-3′ Reverse: 5′-GGGAAGTGGAAGGAAGTGGA-3′	185 bp
KIF20A	Forward: 5′-CGCAGTCACAGCATCTTCTC-3′ Reverse: 5′-GACGAAGGGCAGCAATACAG-3′	202 bp
SPC25	Forward: 5′-TGCAGAGAGGTTGAAAAGGC-3′ Reverse: 5′-TGAGGGGCACTATCTGACAC-3′	198 bp
CHEK1	Forward: 5′-TCAGGTGGTGTGTCAGAGTC-3′ Reverse: 5′-GACATGTGGGCTGGGAAAAG-3′	211 bp
SKA3	Forward: 5′-AGCCCGTAATTGTAACCCCA-3′ Reverse: 5′-TCTGTATCTATGGCCTCCTCAC-3′	211 bp
BUB1	Forward: 5′-TCCCCTCTGTACATTGCCTG-3′	168 bp
	Reverse: 5′-AGCTGGCAAATGGGTTTCAG-3′	
GAPDH	Forward: 5′-TCAAGAAGGTGGTGAAGCAGG-3′ Reverse: 5′-TCAAAGGTGGAGGAGTGGGT-3′	115 bp

**Table 2:** Functional and pathway enrichment analysis of DEGs.

Category	Term	Count	Gene ratio (%)	*P* value
GOTERM_BP	Defense response to gram-negative bacterium	7	1.75%	2.00*E* − 04
GOTERM_BP	Mitotic nuclear division	13	3.24%	5.04*E* − 04
GOTERM_BP	Chromosome segregation	7	1.75%	6.38*E* − 04
GOTERM_BP	Response to yeast	4	1.00%	9.36*E* − 04
GOTERM_BP	Cell division	15	3.74%	1.16*E* − 03
GOTERM_CC	Plasma membrane	95	23.71%	2.95*E* − 05
GOTERM_CC	Specific granule	5	1.25%	3.70*E* − 05
GOTERM_CC	Neuronal cell body	17	4.24%	3.75*E* − 05
GOTERM_CC	Extracellular space	40	9.98%	1.29*E* − 04
GOTERM_CC	Cell surface	20	4.99%	8.98*E* − 04
GOTERM_MF	Carbohydrate binding	9	2.25%	1.09*E* − 02
GOTERM_MF	Peptidase activity	6	1.50%	1.25*E* − 02
GOTERM_MF	Iron ion binding	7	1.75%	3.08*E* − 02
GOTERM_MF	Galactosyltransferase activity	3	0.75%	3.38*E* − 02
GOTERM_MF	Cytokine binding	3	0.75%	3.38*E* − 02
KEGG_pathway	Cell cycle	7	1.75%	1.05*E* − 02
KEGG_PATHWAY	p53 signaling pathway	5	1.25%	1.77*E* − 02
KEGG_PATHWAY	Amoebiasis	5	1.25%	7.41*E* − 02
KEGG_PATHWAY	PPAR signaling pathway	4	1.00%	7.86*E* − 02
KEGG_PATHWAY	Oocyte meiosis	5	1.25%	8.45*E* − 02

## Data Availability

Our data can be found in the GEO database.
